# The Influence of Positive Emotion and Sports Hope on Pre-competition State Anxiety in Martial Arts Players

**DOI:** 10.3389/fpsyg.2020.01460

**Published:** 2020-07-08

**Authors:** HuiXin Yang, XuPing Wen, Fei Xu

**Affiliations:** ^1^College of National Traditional Sports, Harbin Sport University, Harbin, China; ^2^Qiuzhen College, Huzhou University, Huzhou, China

**Keywords:** martial arts, positive emotion, sports hope, path force ideas, emotive force ideas, precompetition state anxiety

## Abstract

**Objectives:**

The present study proposes a model for the relationships among competitive martial arts players’ positive emotions, hope (path and emotive force ideas), sense of control, self-handicapping, and precompetition state anxiety (PCSA). The model seeks to advance our understanding around the causal and mediating effects between these variables and, thus, has important implications for theory and practice in the field of sports and exercise psychology.

**Method:**

A total of 327 participants (male: 255, female: 72; age: 21.45 ± 2.78 years; athletic training: 6.27 ± 2.54 years) in the China University Wushu Sanda Championship 2019 were surveyed. Within 2 h before the match, PCSA, sports hope, sense of control, anxiety, and self-handicapping were measured.

**Results:**

The findings of the present study showed that (1) positive emotions have a significant positive correlation with path and emotive force ideas, anxiety orientation, ability to cope, goal attainment, and sense of control; (2) path and emotive force ideas were significantly positively correlated with anxiety orientation, ability to cope, goal attainment, and sense of control and significantly negatively correlated with anxiety intensity and self-handicapping; and (3) the path idea acted as a partial negative mediator between positive emotion and anxiety intensity, and the sense of control played a partial negative mediator between the emotive force idea and self-handicapping.

**Conclusion:**

(1) Players’ positive emotions can predict the sense of hope. It also tends to interpret the anxiety intensity as a positive challenge rather than a negative sense of control. (2) Players with a higher path idea also tend to have lower negative emotion, anxiety intensity, and self-handicapping; (3) martial arts players’ path idea has a significantly higher predictive power for the sense of control than the emotive force idea. Still, both path and emotive force ideas can indirectly affect the intensity of anxiety orientation and self-handicapping through the multiple mediators of sense of control. Finally, recommendations for coaches related to training and preparation for competition are discussed.

## Introduction

Positive emotion is a critical issue in the field of positive psychology. Individuals with positive emotions will show a more flexible and creative thinking mode. They can find solutions quickly when faced with problems, enabling themselves to have a broad and flexible cognitive style, and then have the ability to integrate various elements (Aliyev and Karakus, 2015; Kim et al., 2019). The sense of hope is a positive motivational state that achieves goals based on the interaction between successful path idea (methods or strategies to achieve goals) and emotive force idea (a goal-oriented emotive force and motivation). Athletes with a higher sense of hope have a stronger motivation for the goal and continuously find ways to achieve their goal so that they will have both higher path and higher emotive force ideas (Bernardo, 2010; Gustafsson et al., 2013). Anxiety refers to the frustration of self-esteem and self-confidence or the increase in feelings of failure and guilt due to the threat of failing to achieve goals or overcome obstacles (Arruda et al., 2014). Anxiety is often regarded as a negative emotion that affects individuals’ performance, and state anxiety is a transitory emotional state consisting of feelings of apprehension, nervousness, and physiological sequelae, such as an increased heart rate or respiration (Smith et al., 2005). However, not all is the same in sports and exercise, and sometimes proper competition anxiety can improve athletes’ performance (Koivula et al., 2002; Hamidi and Besharat, 2010; Jamshidi et al., 2011). A sense of control is an assessment of how much control individuals have in controlling themselves and the environment (Lachman and Weaver, 1998; Chen and Chi, 2012). It includes two dimensions: expectancy of ability to cope and expectancy of goal attainment. Athletes with a sense of control will show a unique ability to cope and expectancy of goal attainment. The state anxiety they generate will promote their athletic performance.

On the contrary, athletes without a sense of control will show negative expectancy ability to cope and the goal attainment. Thus, the state anxiety generated will not be conducive to athletic performance. Self-handicapping is a way for people to make excuses after making mistakes or performing poorly. It is essentially a defensive behavior of individuals against the threat of possible failure. Therefore, self-handicapping is the behavior in which individuals deliberately put themselves in a position that is not conducive to success. When failure occurs, it can be attributed to external control and unstable factors to protect the individuals’ sense of self-esteem or ability. If success happens, it may be attributed to individuals’ achievement and competence (Prapavessis et al., 2003; Kuczka and Treasure, 2005; Ma et al., 2009).

In the field of sports and exercise, people have long been concerned about the causal relationships among variables such as positive emotion, hope, anxiety, control, and self-handicapping (Gustafsson et al., 2010; Aliyev and Karakus, 2015). In terms of positive emotions and hope, many studies support positive emotion as an essential pre-variable of hope (Choe, 2014; Davis et al., 2018; Souza et al., 2018). Athletic competitions will produce different emotional responses, accompanied by the individual’s evaluation of goals, whether they are under pressure, whether they have reached their goals. These continuously produced emotions or moods will cyclically affect path ideas and emotive force ideas (Choe, 2014). When the goal being pursued goes well, positive emotion will be beneficial to enhancing goal pursuit, indicating that the individuals with higher hope enjoy goal pursuits and accelerate the application process of active paths and emotive force ideas; conversely, those with lower hope have different negative emotions, and these negative feelings can lead to self-critical and cognitive problems (Chen and Chi, 2012, 2014, 2015a,b). Their studies have found that athletes had more specific sports goals than the general population. Track-and-field athletes with a higher sense of hope perform significantly better than athletes with lower hope, and regardless of their characteristics or statuses, the sense of hope can significantly predict the actual performance of women’s track-and-field athletes (Tofler et al., 2005); the sense of hope can negatively predict the burnout of competitive athletes (Rasquinha et al., 2014), and the sense of hope of elite athletes can also significantly predict the stress response strategies of problem focus and emotion focus (Chen and Chi, 2009). In the study of sports anxiety, some studies have found that female athletes have significantly higher sports competition anxiety than males (Chen and Chi, 2009; Rasquinha et al., 2014); individual sports athletes have higher precompetition anxiety than team sports athletes (Grossbard et al., 2007) while elite athletes in team sports have higher precompetition anxiety than elite athletes in individual sports. Athletes with 1 year of sports experience have significantly more precompetition anxiety than those with 6 years of sports experience, and young athletes formally selected into the team have lower precompetition state anxiety than the substitute athletes (Zeidner, 1991) while successful athletes have less precompetition state anxiety than less successful athletes (Martinent and Ferrand, 2007; Kaya et al., 2014).

Regarding the sense of control and self-handicapping, Gill et al. (2004) proposed a control model that obstructs and promotes competition anxiety. This model suggests that individuals’ sense of control is affected by differences in sources of stress, which, in turn, affects anxiety orientation. However, the causes of stress are affected by individual differences, so variations in the sense of hope of individuals also affect their subsequent sense of control, which, in turn, affects how individuals interpret anxiety. Athletes may have two different self-handicapping strategies. One is to reduce precompetition efforts, and the other is to make excuses for failure. The present study has found that athletes with higher hope emphasize the best way to face challenges in adversity. When faced with obstacles or frustrations, they only focus on finding breakthroughs and rarely focus on making excuses for failure (Kuczka and Treasure, 2005; Martinent and Ferrand, 2007; Kaya et al., 2014; Chen and Chi, 2015b).

To sum up, the research on hope theory in the sports field is still relatively lacking compared with other fields; especially the empirical research is scarce, and most of the research is to slightly modify the hope scale in the sports field or conduct a cross-sectional inquiry. Second, almost all previous studies considered hope as a single dimension, that is, doing various statistical analyses based on the total score of the hope scale, so it was impossible to know the path or emotive force ideas on the target variables (control, anxiety, and athletic performance) or whether it will have an extraordinary impact. Athletes have more specific sports goals than ordinary people. For example, to improve sports skills or obtain better sports performance, athletes will come up with different methods or strategies for their goals and maintain motivation or driving force. To use these approaches and strategies when the pressure of the game comes, promising athletes may perform better. Based on this, this study combined the theory of hope in positive psychology with related theories of sports psychology and explored the relationship between hope and other sports psychology constructs. That is, the theory of hope was applied to athletes so that this study provides a new direction for the research on sports psychology, and based on the research results, it gives some specific and useful suggestions for sports coaches. Especially for the martial arts players and coaches since the precompetitive state anxiety has a more important role in martial arts competition. Moreover, the research on the precompetitive state anxiety of martial arts players was seldom. To this end, the following hypotheses were proposed: (1) The path and emotive force ideas in the sense of hope are positively correlated to positive emotion, sense of control (i.e., ability to cope and goal attainment), and anxiety orientation but negatively correlated to anxiety intensity and self-handicapping; (2) positive emotion is the pre-variable of sports hope (path and emotive force ideas). Both should influence the anxiety intensity and orientation as well as self-handicapping through the mediating factor of sense of control (i.e., ability to cope and goal attainment).

## Participants and Method

### Participants

China University Wushu Sanda Championship 2019 was held at the Harbin Sport University. The China College Students Sports Association hosted this national-level competition. The questionnaire survey was performed 2 h before the match. The questionnaire contained positive emotion, sports hope, sense of control, precompetition state anxiety, and sports self-handicapping. A relevant database was established to explore the relationship between these variables. A total of 370 athletes with a qualification of being college students participated in the survey. Of them, 341 questionnaires were distributed, and 327 participants in an age range of 19–23 years old were collected of which 255 were males and 72 females. The average age was 21.45 ± 2.78 years, and the average training experience was 6.27 ± 2.54 years.

Before carrying out the survey, the purpose of this study was told to each participant, and a written informed consent form was obtained from each participant. All the procedures followed the local laws and regulations.

### Survey Questionnaire

#### Measurement Instruments

##### The sports hope scale

Using the Chinese version of the Sports Hope Scale compiled by Chen and Chi (2014), the scale contains two subscales with a total of 12 items, of which the path idea subscale includes six items. The emotive force idea subscale consists of six items. Using an 8-point scale, from 1 to 8 represents “extremely disagree” to “extremely agree,” respectively.

The items on the scale are listed as follows:

Emotive force idea: (1) I have a strong motivation to pursue my sports goals, (2) I actively pursue my sports goals, (3) I will pursue my goals with practical actions instead of just dreaming, (4) I think there is a motive force behind me to push me to my sports goals, (5) the emotive force to reach my sports goals makes me continuously think about how to break through, (6) I would achieve the sports goals I set for myself.

Path idea: (1) I can come up with many ways to get out of trouble during sports competitions; (2) I can come up with many ways to deal with any problem in sports; (3) I can come up with many strategies to achieve my sports goals; (4) when the initial competition strategy does not work, I can think of other plans; (5) I can think of some strategy to reverse the worsening trend during sports competitions; (6) in practice, even if others are discouraged, I know I still can find a solution to the problem.

##### The positive emotion coping scale

The Chinese version of the emotional response scale compiled by Li et al. (2017) was used to measure the emotional response of the players. It contains 10 entries. The emotion intensity was measured with a 5-point scale, from 1 to 5 for “no feeling” to “powerful feeling,” respectively.

The items of the scale are listed as follows:

Your feelings in the past week were (1) interested, (2) excited, (3) firm, (4) enthusiastic, (5) proud, (6) motivated, (7) determined, (8) active, (9) alert, (10) flexible.

##### The sense of control scale

Based on the control scale by Roberts and Robert Ho (1996), the scale has been revised, including a total of 9 entries. The scale contained two subscales. It was measured with a 4-point scale. From 1 to 4 represents “extremely disagree” to “extremely agree,” respectively. Finally, the scores of each entry were added up. The expectancy of ability to cope subscale contained eight items, and the expectancy of goal attainment scale contained two items. The two entries of the goal attainment test were designed as follows: question 1: “whether to set the goal to win before the match” and question 2: “whether to set the goal of the attained performance before the match.” If the response to the first question ticked “Yes,” then the participant was required to choose the degree of goal attainment in the second question. The score range of question 2 was 1–7 points. The higher the score was, the higher the score of goal attainment tended to be. If the participant selected “No” in the first question, it meant that the participant had not set a sports goal and was recorded as 0 points.

The items of the scale are listed as follows:

Ability to cope subscale:(1) As long as I make up my mind, no technical and tactical skills cannot be learned by me; (2) the achievements I have made in the game are entirely based on my diligence and intelligence; (3) as long as I set the goal, in most cases I can reach it; (4) most things that happen in my life are beyond my control; (5) it does not make sense for me to spend too much effort on difficult situations; (6) once I have a plan, I am almost sure I can achieve it; (7) sometimes bad luck prevents me from what I want to accomplish; (8) when I make my efforts, I usually perform what I want to achieve.

Goal attainment: (1) Do you set a goal to win the game before the competition? (2) Your goal attainment for this game is (You can choose 1–7, 1 for “no confidence” and 7 for “absolute confidence”).

##### The precompetition state anxiety scale

This study was mainly based on the precompetition state anxiety scale by Hanton et al. (2002) and Martinent et al. (2010), which contained 12 entries, of which 6 were precompetition cognitive anxiety entries (for example, “I worry about poor performance”) and physical anxiety before competition was six entries (for example, “My heartbeat increases rapidly”). In addition to measuring the intensity of anxiety symptoms before a competition, this scale also measured the anxiety orientation. In measuring the intensity of anxiety, a 4-point scale was adopted. From 1 to 4 represented “strongly disagree” to “strongly agree,” respectively. The higher the score was, the higher the intensity of cognitive or physical anxiety tended to be. For the orientation measurement, the range was from −3 to +3 for whether they were favorable or harmful: The −3 meant that it was not suitable for performance; 0 meant that it did not affect performance; +3 meant that it was perfect for performance. The lower the total score the participant has, the more the participant regarded anxiety as a detriment to performance. In contrast, the higher the total score the participant got, the more the participant explained anxiety as a benefit to performance.

The items of the scale are listed as follows:

Physical anxiety: (1) I feel nervous, (2) I have a tense stomach, (3) my heart beats fast, (4) I feel a drooping belly, (5) My palms are sweating, (6) I feel tense throughout my body.

Cognitive anxiety: (1) I worry about not being able to do my best in the game, (2) I worry about losing the game, (3) I worry about poor performance, (4) I worry about whether I can achieve my goal, (5) I worry about other people being disappointed with my performance, (6) I fear that I cannot focus on the game.

##### The sports self-handicapping scale

Using Wu et al. (2004) one-dimensional sports self-handicapping scale, which includes a total of 14 questions, a 5-point scale was adopted, 5 represented “always,” and 1 represented “never.”

The items of the scale are listed as follows:

(1) When my sports performance is poor, I often think that it is the presence of others that affects me, (2) I always start to practice exercise test items at the last minute, (3) I often feel weak and inadequate in sports activities, (4) I will try my best to learn any difficult sports skills, (5) I will easily affect my performance due to environmental factors, (6) I will deliberately not take seriously competitive sports activities in case of poor performance making me lose face, (7) If I work harder on sports skills, I will perform better, (8) sometimes feeling sick or injured is also good because this can just lift some sports stress during a test or competition, (9) if my mood can be stabilized a bit, I should have a better sports performance, (10) when I live up to expectations of others in sports activities, I will try to seek reasonable explanations, (11) I feel that I am less lucky than others in many sports tests or competitions, (12) someday I will start to work hard on sports skills, (13) because of some others’ ideas, I often cannot focus on learning motor skills, (14) sometimes I think my mood is very depressed, thus making some of the original easy-to-follow movements more difficult.

#### Validity and Reliability Test of the Questionnaire

Table 1 shows:

**TABLE 1:** Quality analyses of four measurement scales.

**Scales**	**Dimension naming**	**KMO & Bartlett test**	**Entries**	**Explained variance (%)**	**Accumulated explained (variance) %**	**Combined confidence CR**	**Cronbach α**
		P = 0.000					
Hope for sports scale	Path	KMO = 0.89 *p* < 0.05	6	34.16	34.16	0.87	0.79
	Emotive force		6	24.25	58.41	0.86	0.84
Positive emotion scale	Positive emotion	KMO = 0.87; *p* < 0.05	10	75.89	75.89	0.81	0.88
Sense of control scale	Ability to cope	KMO = 0.83 *p* < 0.05	8	57.69	57.69	0.84	0.89
	Goal attainment		2	*r* = 0.76^1^			
Precompetition	Physical	KMO = 0.89 *p* < 0.05	6	31.24	31.24	0.84	0.87
state anxiety scale	Cognitive anxiety		6	22.47	53.71	0.81	0.83
Self-handicapping scale	Uni-dimension	KMO = 0.87 *p* < 0.05	14	57.17	57.17	0.81	0.86
			–	–	–	–	–

*^1^This dimension only needs to be test-retested.*

(1) Exploratory factor analysis of hope for the sports scale consisting of 12 questions showed that it was very suitable for factor analysis (KMO = 0.89; *p* < 0.05). It could extract two common factors (named path factor and emotive force factor), and their explanatory powers were 34.16 and 24.25%, respectively, with an accumulated contribution rate of 58.41%. In terms of reliability, the Cronbach’ α coefficients of the two factors were 0.79 and 0.84, respectively, and the overall α coefficient was 0.80; the verified factors showed that the measurement model fitness indices of AGFI, CFI, NFI, and IFI were 0.93, 0.95, 0.94, and 0.93, respectively. All values were greater than the criteria of 0.90, RMSEA = 0.037 (less than 0.05 indicating a good fit). Besides, the combined reliability CR of the two factors (latent variables) was 0.87 and 0.86, which showed that the reliability and validity of this scale were excellent.
(2) Only a common factor (KMO = 0.87; *p* < 0.05) was extracted from a 10-question positive emotive scale, and the explanatory power was 75.89%. In terms of reliability, the Cronbach’s α coefficient of the overall scale was 0.88. In the test of the fitness of the measurement model, it showed that AGFI = 0.91, CFI = 0.95, NFI = 0.92, and IFI = 0.93, all of which were greater than the criteria of 0.90, and RMSEA = 0.035 (less than 0.05 indicating a good fit). The combined reliability CR was 0.81, which showed that the reliability and validity of this scale were good.
(3) The sense of control scale contained two dimensions; the first dimension contained eight items. After exploratory factor analysis (KMO = 0.83; *p* < 0.05), a common factor could be extracted, which was named “ability to cope,” and the contribution rate was 57.69%. In terms of reliability, the Cronbach’s α coefficient of this dimension was 0.89; the fitness indices of the measurement model of AGFI, CFI, NFI, and IFI, were 0.90, 0.93, 0.94, and 0.92, respectively, all of which were greater than the criteria of 0.90. RMSEA = 0.042 (less than 0.05 indicating a good fit); the combined reliability CR of the latent variables in this dimension was 0.84. For dimension 2, because of its distinctive design, only the retest reliability test was needed, and the correlation coefficient was *r* = 0.76. It showed that the reliability and validity of the sense of control scale were excellent.
(4) Exploratory factor analysis of the precompetition anxiety scale with 12 questions showed that it was very suitable for factor analysis (KMO = 0.89; *p* < 0.05). It could extract 2 common factors (named physical anxiety and cognitive anxiety), and their explanation powers were 31.24 and 22.47%, respectively, with an accumulated contribution rate of 53.71%. In terms of reliability, the Cronbach’s α coefficients of the two factors were 0.87 and 0.83, respectively, and the overall α coefficient was 0.88; the measurement model fitness indices of AGFI, CFI, NFI, and IFI were 0.92, 0.95, 0.90, and 0.93, respectively, all of which were greater than the criteria of 0.90, RMSEA = 0.032 (less than 0.05 indicating a good fit). Besides, the combined reliability CR of the two latent variables were 0.84 and 0.81, respectively, which showed that the scale had excellent reliability and validity.
(5) The sports self-handicapping scale with 14 entries could only extract one common factor (KMO = 0.87; *p* < 0.05), and its explanatory power was 57.17%. In terms of reliability, the Cronbach’s α coefficient of the overall scale was 0.86. In the test of the fit of the measurement model, it showed that AGFI = 0.94, CFI = 0.92, NFI = 0.93, and IFI = 0.95, all of which were greater than the criteria of 0.90, and RMSEA = 0.036 (less than 0.05 for a good fit). The combined reliability CR was 0.81, which showed that the reliability and validity of this scale were good.

### Statistical Analysis

**FIGURE 1:**
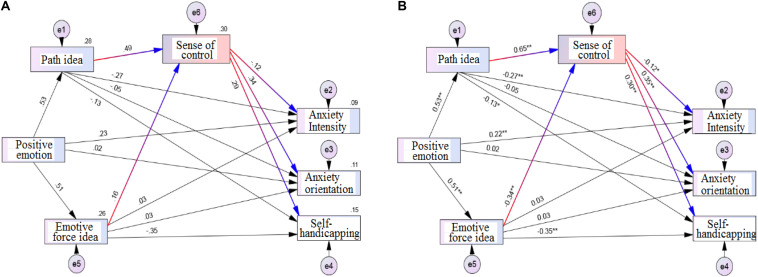
Architecture diagram of the theory of hope and the sense of control of precompetition state anxiety model: **(A)** initial model; **(B)** modified model.

After deleting the uncompleted and invalid questionnaires, the database has been established. Using SPSS 21.0 and Amos 21.0 software as processing and analysis tools, the Pearson cross-product correlation was used to explore the correlation levels of each variable. It then used exploratory factor analysis (EFA) combined with confirmatory factor analysis (CFA) to verify the hypotheses of this study; the significance level of all tests was set at α = 0.05. The architecture diagram of the theory of hope and the sense of control of precompetition state anxiety model have been established and modified (Figure 1).

## Results

### Normality Test of Various Variables

Table 2 shows that the skewness values of the scores of 9 variables, such as positive emotions, path ideas, emotive force ideas, anxiety intensity, anxiety orientation, self-handicapping, ability to cope, goal attainment, and sense of control ranged from 0.39 to 1.41. In the meantime, the kurtosis values ranged from 0.38 to 1.27, which indicated that the sample scores of the 9 variables satisfied the necessary conditions of normal distribution.

**TABLE 2:** Normality distribution test of all nine variables.

**Variable**	**Skewness**	**Kurtosis**	**Distribution**
Positive emotion	–0.59	–0.38	Normality
Path ideas	–0.85	–0.71	Normality
Emotive force ideas	0.73	0.48	Normality
Anxiety intensity	–0.39	–0.79	Normality
Anxiety orientation	1.03	1.27	Normality
Selfhandicapping	–0.55	–0.81	Normality
Ability to cope	1.22	–0.88	Normality
Goal attainment	1.41	–0.87	Normality
Sense of control	0.91	–0.44	Normality

### Correlation Analysis Across Various Variables

Table 3 shows (1) The relationships of positive emotions with path ideas (0.53^∗∗^), emotive force ideas (0.51^∗∗^), anxiety orientation (0.18^∗∗^), expectancy of ability to cope (0.47^∗∗^), expectancy of goal attainment (0.41^∗∗^), and the sense of control (0.50^∗∗^) showed significant positive correlations, which meant that the more positive emotions the individuals have, the higher the emotive force and path ideas tended to be and the better the sense of control would be, and these tended to interpret anxiety as the favorable variable. (2) The relationship of path idea with emotive force (0.72^∗∗^), anxiety orientation (0.18^∗∗^), expectancy of ability to cope (0.55^∗∗^), expectancy of goal attainment (0.47^∗∗^), and sense of control (0.58^∗∗^) were significantly positively correlated (*p* < 0.01) and significantly negatively correlated with anxiety intensity (−0.20^∗∗^) and self-handicapping (−0.21^∗∗^) (*p* < 0.01), which meant that the higher the individual’s path idea was, the higher the tendency of the emotive force idea, and sense of control tended to interpret anxiety as a favorable orientation, but it also tended to lower the intensity of anxiety and self-handicapping.

**TABLE 3:** Matrix of correlation coefficient of various variables.

	**PE**	**PI**	**EFI**	**AI**	**AO**	**SH**	**EAC**	**EGA**	**SC**
PE	1.00	−	−	−	−	−	−	−	−
PI	0.53**	1.00	−	−	−	−	−	−	−
EFI	0.51**	0.72**	1.00	−	−	−	−	−	−
AI	0.04	−0.20**	–0.11	1.00	−	−	−	−	−
AO	0.18**	0.18**	0.17**	−0.25**	1.00	−	−	−	−
SH	–0.07	−0.21**	−0.30**	0.34**	–0.08	1.00	−	−	−
EAC	0.47**	0.55**	0.41**	−0.28**	0.30**	0.08	1.00	−	−
EGA	0.41**	0.47**	0.44**	–0.00	0.16*	0.06	0.43**	1.00	−
SC	0.50**	0.58**	0.49**	–0.15	0.34**	0.05	0.70**	0.82**	1.00
Mean	3.45	5.55	5.91	2.58	–0.28	2.35	2.40	4.68	6.62
Deviation	0.89	0.79	0.95	0.69	0.98	0.78	0.71	1.41	1.82

PE, positive emotion; PI, path idea; EFI, emotive force idea; AI, anxiety Intensity; AO, anxiety orientation; SH, Self-handicapping; EAC, expectancy of ability to cope; EGA; expectancy of goal attainment; SC, sense of control. *p < 0.05; **p < 0.01.

### Hypothesis Association Model Analysis

In the architecture diagram of the theory of hope and the sense of control of the precompetition state anxiety model proposed in this study, it was assumed that positive emotions would predict the path and emotive force idea positively. Path and emotive force ideas would predict the sense of control (i.e., ability to cope and goal attainment). The sense of control could predict the intensity, orientation, and self-handicapping of precompetition state anxiety.

#### Measurement and Verification of Structural Models

Table 4 shows (1) From the results of the absolute fit test: the absolute fit index of the initial model: *X*^2^ = 90.95, *X*^2^/*df* = 11.24, *p* = 0.000 < 0.05, indicating that the covariance matrix of the hypotheses model did not fit well with the observed data (Generally, the *X*^2^/*df* value should be between 1 and 3 for good fit); GFI = 0.843 (>0.90 for good fit), AGFI = 0.767 (>0.90 for good fit), RMSEA = 0.346 (generally, RMSEA < 0.05 for excellent, 0.05 to 0.08 for good). From the results of the value-added fit test, NFI = 0.659 (>0.90 for good fit), IFI = 0.664 (>0.90 for good fit), CFI = 0.657 (>0.90 for good fit), RFI = −0.195 (>0.90 for good fit). In short, whether it was an absolute or value-added fit test, the initial correlation model of this study did not fit well with the observed data, so the correlation model must be revised.

**TABLE 4:** Test of the degree of fit of models.

**Model**	**Absolute fit**						**Value-added fit**			
	***X*^2^**	***X*^2^/*df***	***p*-value**	**RMSEA**	**GFI**	**AGFI**	**NFI**	**IFI**	**CFI**	**RFI**
Initial model	90.95	11.24	0.000	0.346	0.843	0.767	0.659	0.664	0.657	–0.195
Modified model	4.76	2.38	0.092	0.065	0.992	0.942	0.993	0.996	0.996	0.929

**p* < 0.05 indicates that the model fits poorly; *p* > 0.05 indicates that the model fits well.*

(2) In this study, based on the path suggested by the correction index in the initial model, the original model was modified in accordance with the original theoretical framework, and the covariance relationship between the error items of the measurement index (e1-e5, e2-e4, e2-e3, e2-positive emotions). The results showed that the absolute fit index of the revised model was *X*^2^ = 4.76, *X*^2^/*df* = 2.38, and *p* = 0.092 > 0.05, indicating that the covariance matrix of the hypothetical model was fit to the observed data (*X*^2^/*df* = 2.38, which was between 1 and 3 for good fit); GFI = 0.992 (>0.90 for good fit), AGFI = 0.942 (>0.90 for good fit), and RMSEA = 0.065 (from 0.05 to 0.08 for good). From the results of the value-added fit test, NFI = 0.993 (>0.90 for good fit), IFI = 0.996 (>0.90 for good fit), CFI = 0.996 (>0.90 for good fit), RFI = 929 (>0.90 for good fit). After revision of the initial correlation model, it achieved a better fit with observed data.

#### Path Analysis of the Influence of Positive Emotion and Hope on Precompetition Anxiety

Figure 1B and Table 5 show (1) from the perspective of direct effect, standardized path coefficients indicated that positive emotions had significant direct effects on path ideas (*R* = 0.53^∗∗^) and positive emotions on the emotive force idea (*R* = 0.51^∗∗^). Emotive and path force ideas also had significant direct effects on the sense of control (*R* = −0.34^∗∗^, 0.65^∗∗^, respectively). The sense of control had a direct and significant influence on the intensity of anxiety (*R* = −0.12^∗^), the anxiety orientation (*R* = 0.35^∗∗^), and self-handicapping (*R* = 0.30^∗∗^).

**TABLE 5:** The direct and indirect effect of path idea, emotive force idea, and sense of control on anxiety state and self-handicapping.

**Variables**	**Positive emotion**			**Emotive force**			**Path idea**			**Sense of control**		
	**Direct effect**	**Total effect**	**Indirect effect**	**Direct effect**	**Total effect**	**Indirect effect**	**Direct effect**	**Total effect**	**Indirect effect**	**Direct effect**	**Total effect**	**Indirect effect**
EFI	0.51**	0.51**	0.00	0.00	0.00	0.00	0.00	0.00	0.00	0.00	0.00	0.00
PI	0.53**	0.53**	0.00	0.00	0.00	0.00	0.00	0.00	0.00	0.00	0.00	0.00
SC	0.00	0.17*	0.17*	−0.34**	−0.34**	0.00	0.65	0.65	0.00	0.00	0.00	0.00
SH	0.00	−0.20**	−0.20**	−0.35**	−0.46**	−0.10*	−0.13*	0.07*	0.19**	0.30*	0.30*	0.00
AO	0.02	0.07	0.05	0.03	−0.09*	−0.12	–0.05	0.18*	0.23**	0.35*	0.35*	0.00
AI	0.22**	0.07	−0.15*	0.03	0.07	0.04	−0.27*	−0.35*	−0.08*	−0.12*	−0.12*	0.00

PI, path idea; EFI, emotive force idea; AI, anxiety Intensity; AO, anxiety orientation; SH, self-handicapping; SC, sense of control. *p < 0.05; **p < 0.01.

(2) Positive emotions could directly affect the intensity of anxiety (*R* = 0.22^∗∗^) and could also negatively affect the intensity of anxiety indirectly through the path idea as a partial mediator (*R* = −0.15^∗^). The emotive force idea could also directly affect the self-handicapping. The direct effect *R* = −0.35^∗∗^. It can also indirectly and negatively affect the self-handicapping through the partial mediating role of a sense of control. The indirect negative effect was *R* = −0.10^∗^; meanwhile, the emotive force idea could also influence the anxiety orientation through the full mediator of sense of control with a negative effect *R* = −0.12^∗^. The path idea could indirectly affect the anxiety orientation through the full mediation of the sense of control, and its positive effect was *R* = 0.23^∗∗^, or it could indirectly affect the self-handicapping through the mediation of the sense of control, and its positive effect was *R* = 0.19^∗∗^; meanwhile, the path idea could also indirectly and negatively affect the intensity of anxiety through the mediator of the sense of control, and its negative effect was *R* = −0.08^∗^.

## Discussion

This research combined the theory of hope with the related theories of sports psychology, especially the precompetition state anxiety control model, and put forward the association model hypothesis. As a result, it has found that after the model was modified, whether it was absolute or value-added tests, both tests suggested that the correlation pattern in this study had fit well with the observed data. Positive emotions have been expressed as the pre-variable of sports hope (i.e., path idea and emotive force idea), which affected the intensity, orientation, and self-handicapping of anxiety via multiple mediating factors of the sense of control (i.e., ability to cope and goal attainment).

### From the Relationship Between Positive Emotion, Hope, and Control

The correlation coefficient matrix in Table 3 revealed that both path and emotive force ideas were significantly positively related to positive emotion, anxiety orientation, and sense of control. The higher the positive emotion was, the higher the path and emotive force tended to be, and also the higher the sense of control tended to be, and the tendency to explain anxiety as beneficial factor to athletic performance. The path idea was significantly negatively related to anxiety intensity and self-handicapping, indicating that the higher the path idea was, the lower the anxiety intensity and self-handicapping tended to be. The emotive force idea was significantly negatively related to self-handicapping, indicating that the higher the emotive force idea, the lower the self-handicapping tended to be. However, in this study, the correlation coefficient between the emotive force idea and anxiety intensity (*r* = −0.11) did not reach a significant negative correlation level, which was different from that reported by Chen and Chi (2015b). The result may be because the level of the competition was not high; the goal of most of the participating teams were to train newcomers (most of the players were second-tier athletes). Although they were motivated (emotive force idea), they were still prone to anxiety due to a lack of experience in the competition. Therefore, there was no significant negative correlation between the emotive force idea and anxiety intensity. However, it was still negatively correlated from the correlation coefficient, so it still had some statistical significance. Besides, because the male and female ratio was too different in this study (255 males and 72 females), no gender differences were explored for variables such as positive emotion, hope, and control.

### From the Effect of Sports Hope on the Sense of Control

In previous research, Chen and Chi (2015a) found that middle-aged people’s emotive force idea could more positively predict problem-solving than the path idea, which affected psychological adjustment (including depression and life satisfaction). Retnowati et al. (2015) found that only the emotive force idea could negatively predict anxiety and depression 1 month later. Du and King (2013) explored the relationship between hope and goal attainment and found that the emotive force idea had a higher predictive power for goal achievement than the total score of hope. In short, after separating the path idea from the emotive force idea in the previous research, the results showed that the influence of the emotive force idea was stronger than the path idea. However, this study found that, from the average of the path and emotive force ideas, there was almost no difference between the path idea (*M* = 5.55) and emotive force idea (*M* = 5.91), which was consistent with previous studies that analyzed path and emotive force ideas mean scores through separated the sense of hope (Jamshidi et al., 2011; Chen and Chi, 2014; Laborde et al., 2017; Woodgate et al., 2017). This study appeared to be different from previous ones when predicting the impact of subsequent outcome variables. The results of this study indicated that the effect of path ideas was stronger than that of emotive force idea, which was manifested by the influence of path idea on the sense of control (*R* = 0.65^∗∗^) and the direct impact on the intensity of anxiety (*R* = −0.27^∗∗^) while the influence of emotive force ideas on the sense of control (*R* = −0.34 ^∗∗^) had almost no effect on the intensity of anxiety (*R* = 0.03). How to explain this discrepancy between the results of this study and previous studies? According to the original hypothesis of the theory of hope, path and emotive force ideas were equally important to hope. The higher hope represents the higher path and emotive force. However, if the sense of hope was separated to discuss its impact on subsequent results, this may make a difference in statistics. This study found that the predictive power of path ideas for sense of control was significantly higher than that of emotive force idea. For a sense of control, the influence of path ideas may be more critical than emotive force ideas. Compared with the emotive force idea, an athlete with a higher path idea will continuously think about how to reach the goal. When he comes up with many methods or strategies to achieve the goal, there may be a relatively high sense of control. This result seems to imply that, in the future research on hope, it is necessary to continue to explore the relationship between path ideas and emotive force ideas on different outcome variables. Only in this way can we indeed reveal the construction of hope theory and better understand the theory of hope and its implications as well as its relationship between subsequent outcome variables.

### From the Influence of Multiple Mediators of Sense of Control

The present study found that positive emotions can directly affect the intensity of anxiety. Still, it can also indirectly negatively affect the intensity of anxiety by using path ideas as a partial intermediary (intermediate influence *R* = −0.15^∗^). Again, positive emotions cannot indirectly change the intensity of anxiety and the anxiety orientation via the emotive force idea. Both the path and emotive force ideas can have a direct negative influence on self-handicapping, but the direct influence of the path idea is lower than emotive force idea (*R* = −0.13^∗^, −0.35^∗∗^). Whether it is a path idea or emotive force idea, both can use the sense of control as a mediator to influence precompetition anxiety, but the mediating effect of the path idea is significantly higher than that of the emotive force idea. It is manifested that the emotive force idea can only affect both the anxiety orientation and self-handicapping. However, the path idea can influence anxiety intensity, orientation, and self-handicapping through the sense of control (intermediary), and the mediating effect also shows that the path idea is significantly higher than the emotive force idea (0.19^∗^ and 0.28^∗^ vs. −0.10^∗^ and −0.12^∗^). These results further validate the hypothesis of this study that positive emotions are used as a pre-variable of path ideas and emotive force ideas, in which path and emotive force ideas can influence the intensity of anxiety, orientation, and self-handicapping through the intermediary of sense of control. Besides, paths and emotive force ideas are significantly negatively related to self-handicapping (*r* = −0.21^∗∗^, −0.30 ^∗∗^), which is in line with previous scholars’ opinions (Arruda et al., 2014; Chen and Chi, 2015a; Kim et al., 2019). Individuals with higher hope will focus on finding breakthroughs when faced with obstacles or setbacks, and it is unlikely to reduce efforts or make excuses. It also accords with the viewpoint that the way of thinking may be opposite to reason; that is, athletes with high hopes should hardly make excuses (Gustafsson et al., 2013).

### The Limitations of the Present Study

Owing to the gender imbalance (male 255, female 72, Chi-squared test: χ^2^ = 102.4, *p* < 0.001), the present study took both female and male as an overall population. Sex differences may exist, which will be carried out in future research.

In this questionnaire, owing to using the original rating scale, an 8-point scale was used to measure sports hope. This scale is rarely used in research, partly because it is easier for respondents to use either a 10-point scale or between 5- and 7-point scales. A 5-point scale was used to measure the positive emotion coping, and a 4-point scale was used to measure the sense of control. The goal attainment had a 7-point scale, and anxiety was measured on a 4-point scale. Different scales were used within the same questionnaire for the different items. It might make a confusing effect on the participants’ ratings. In future research, the rating scale should be united into a 5 or 7 points in the same questionnaire.

## Conclusion

(1) Athletes’ positive emotions can predict a sense of hope. The higher the path idea scores, the higher the positive emotion, emotive force idea, and sense of control tend to be. They also tend to interpret anxiety as a positive challenge rather than an uncontrollable negative feeling. (2) Athlete’s path ideas have a significantly higher predictive power for the sense of control, and martial arts players with higher path ideas also tend to have lower negative emotions, anxiety intensity, and self-handicapping. (3) The role of sense of control as multiple intermediary identities are established; both path and emotive force ideas can influence the precompetition state anxiety through the sense of control. However, the in-depth influence of path idea is significantly higher than those of emotive force idea.

## Suggestions and Further Research Orientation

### Suggestions for Coaches

1) Positive emotion is a very important pre-variable of hope theory. Coaches should try to create a pleasant training environment and appropriate motivational climate for inducing athletes’ positive emotions in practice or precompetition, thereby strengthening the player’s sense of hope to achieve the goal of improving sports performance.
2) If the player finds that the path idea is low, the coach can increase his goal-oriented thinking, let the player set the goal, and let him think about how to achieve the goal in each practice or competition. If motivation is relatively low, we should pay attention to the training of the motivation part of the players and encourage them to create a climate of motivation to achieve their goals.
3) The sense of hope can affect the intensity of anxiety, orientation, and self-handicapping through the multiple mediators of sense of control, so the coach can try to make the players physically successful, guide the players to think positively through the precompetition simulation training, encourage the athletes to maintain self-confidence, and develop the habit of finding ways to achieve their goals, thereby increasing the sense of control.
4) The present study is benefical for the martial arts players and coaches since the precompetitive state anxiety has a more important role on martial arts competition.

### Future Research Direction

1) This study is a cross-sectional study. In the future, the long-term relationship between hope, control, and precompetition anxiety can be explored experimentally. At the same time, to what extent does the increase in sports hope affect anxiety intensity, orientation, and self-handicapping? In the future, long-term, continuous research should be used to verify the long-term effects of the sense of hope on negative psychological variables.
2) There are considerable differences in the training background, sports motivation, and sports skills of the subjects in this study, which may have an impact on the hypothetical model in this study. Future studies can choose different sports events players to continue to explore the generality of the model and continue to distinguish the influence of path and emotive force ideas on different post-variables further to improve the construct of hope theory and expand its application field.

## Data Availability Statement

All datasets presented in this study are included in the article/supplementary material.

## Ethics Statement

Ethical review and approval was not required for the study on human participants in accordance with the local legislation and institutional requirements. The patients/participants provided their written informed consent to participate in this study.

## Author Contributions

All authors designed the study, processed the data, and revised the manuscript. HY and FX performed the survey. HY and XW wrote the first draft.

## Conflict of Interest

The authors declare that the research was conducted in the absence of any commercial or financial relationships that could be construed as a potential conflict of interest.
